# [(1,2,5,6-η)-Cyclo­octa-1,5-diene]bis­(1-isopropyl-3-methyl­imidazolin-2-yl­idene)rhodium(I) tetra­fluorido­borate

**DOI:** 10.1107/S1600536811049890

**Published:** 2011-11-30

**Authors:** Gary S. Nichol, Jonathan Rajaseelan, David P. Walton, Edward Rajaseelan

**Affiliations:** a Department of Chemistry and Biochemistry, The University of Arizona, Tucson, AZ 85716, USA; b Lancaster Country Day School, Lancaster, PA 17603, USA; c Department of Chemistry, Millersville University, Millersville, PA 17551, USA

## Abstract

In the title compound, [Rh(C_8_H_12_)(C_7_H_12_N_2_)_2_]BF_4_, the square-planar Rh complex cation and the BF_4_^−^ anion are both bis­ected by a crystallographic twofold rotation axis. The Rh and B atoms lie on this axis and all others are in general positions. In the crystal, two unique C—H⋯F hydrogen-bonding inter­actions are present, which involve both imidazolin-2-yl­idene H atoms. They form two separate *C*(5) motifs, the combination of which is a rippled hydrogen-bonded sheet structure in the *ab* plane.

## Related literature

For the structure and dynamics of related *N*-heterocyclic carbene rhodium and iridium complexes, see: Chianese *et al.* (2003 ▶); Köcher & Herrmann (1997 ▶); Leung *et al.* (2006 ▶); Nichol *et al.* (2009 ▶, 2010 ▶); Herrmann *et al.* (2006 ▶). For the catalytic properties of these complexes, see: Albrecht *et al.* (2002 ▶); Frey *et al.* (2006 ▶); Gnanamgari *et al.* (2007 ▶); Voutchkova *et al.* (2008 ▶).
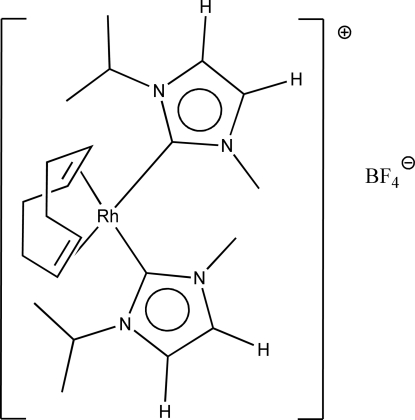


## Experimental

### 

#### Crystal data

[Rh(C_8_H_12_)(C_7_H_12_N_2_)_2_]BF_4_
*M* *_r_* = 546.27
Orthorhombic, 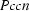

*a* = 11.7508 (6) Å
*b* = 11.9283 (6) Å
*c* = 17.3129 (9) Å
*V* = 2426.7 (2) Å^3^
*Z* = 4
Mo *K*α radiation
μ = 0.75 mm^−1^
*T* = 100 K
0.38 × 0.37 × 0.37 mm

#### Data collection

Bruker Kappa APEXII DUO CCD diffractometer
Absorption correction: multi-scan (*SADABS*; Sheldrick, 1996 ▶) *T*_min_ = 0.763, *T*_max_ = 0.771
234794 measured reflections
14018 independent reflections
10241 reflections with *I* > 2σ(*I*)
*R*_int_ = 0.033

#### Refinement

*R*[*F* ^2^ > 2σ(*F* ^2^)] = 0.020
*wR*(*F* ^2^) = 0.059
*S* = 1.13
14018 reflections
218 parameters
All H-atom parameters refined
Δρ_max_ = 1.55 e Å^−3^
Δρ_min_ = −0.92 e Å^−3^

### 

Data collection: *APEX2* (Bruker, 2007 ▶); cell refinement: *SAINT* (Bruker, 2007 ▶); data reduction: *SAINT*; program(s) used to solve structure: *SHELXTL* (Sheldrick, 2008 ▶); program(s) used to refine structure: *SHELXTL*; molecular graphics: *SHELXTL* and *Mercury* (Macrae *et al.*, 2006 ▶); software used to prepare material for publication: *SHELXTL* and *publCIF* (Westrip, 2010 ▶).

## Supplementary Material

Crystal structure: contains datablock(s) I, global. DOI: 10.1107/S1600536811049890/fj2478sup1.cif

Structure factors: contains datablock(s) I. DOI: 10.1107/S1600536811049890/fj2478Isup2.hkl

Supplementary material file. DOI: 10.1107/S1600536811049890/fj2478Isup3.cdx

Additional supplementary materials:  crystallographic information; 3D view; checkCIF report

## Figures and Tables

**Table 1:** Hydrogen-bond geometry (Å, °)

*D*—H⋯*A*	*D*—H	H⋯*A*	*D*⋯*A*	*D*—H⋯*A*
C2—H2⋯F1^i^	0.909 (11)	2.496 (11)	3.3975 (8)	171.4 (10)
C3—H3⋯F2^ii^	0.877 (12)	2.478 (12)	3.2415 (8)	145.9 (11)

Symmetry codes: (i) 
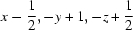
; (ii) 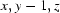.

**Fig. 1.**
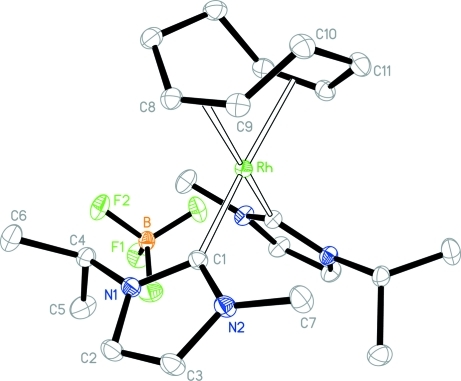
Twice the asymmetric unit of (I), with H atoms omitted. Displacement ellipsoids are at the 50% probability level. Unlabeled atoms are related to labeled atoms by twofold rotation symmetry.

**Fig. 2.**
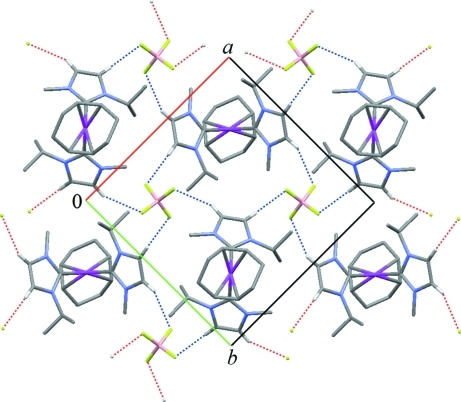
A c-axis projection showing C–H···F interactions (blue dotted lines) in (I). Red dotted lines indicate H bond continuation.

## supplementary crystallographic information

### Comment

We are interested in rhodium and iridium complexes with N-heterocyclic carbene ligands, in particular ligands derived from 1,2,4-triazole-derived compounds (Nichol *et al.*, 2009, 2010). The title compound, (I), was prepared as part of this study (Figure 1). The Rh center has an expected square planar geometry and bond distances are unexceptional. Both the Rh and B atoms lie on a crystallographic twofold rotation axis, which bisects the complex and BF_4_^-^ counterion. C–H···F hydrogen bonding interactions, which involve both imidazolin-2-ylidene H atoms and all four F atoms, form a thick two-dimensional sheet structure in the *ab* plane (Figure 2).

### Experimental

The title compound was synthesized by transmetallation. 1-Isopropyl-3-methylimidazolium bromide (268 mg, 1.31 mmol) was mixed with Ag_2_O (152 mg, 0.654 mmol), and was stirred under dark at room temperature for 90 minutes in 10 ml of CH_2_Cl_2_. The resulting mixture was filtered through Celite into a new flask containing the neutral compound [(cod)Rh(NHC)Cl](585 mg, 1.31 mmol), and AgBF_4_(254 mg, 1.31 mmol)and stirred for an additional 90 minutes under dark. The mixture was filtered once more through Celite to remove silver bromide and silver chloride, and the solvent was removed under pressure to give a yellow solid (93%). Crystals of the resulting solid of the title compound, (I), were obtained by slow diffusion of pentane into dichloromethane solution of the compound. ^1^H NMR (400 MHz, CDCl_3_): δ (p.p.m.) = 7.15 (s, 2 H, NC*H*), 6.93 (s, 2 H, NC*H*), 5.03 (m, ^3^J_H—H_ = 6.8 Hz, 2 H, CH of ^i^Pr), 4.63 (br, 2 H, CH of COD), 4.21 (s, 6 H, N—CH_3_), 3.92 (m, 2 H, CH of COD), 2.63 (m, 2 H, CH_2_ of COD), 2.42 – 1.92 (m, 6 H, CH_2_ of COD), 1.46 (d, ^3^J_H—H_ = 6.8 Hz, 6 H, CH_3_ of ^i^Pr), 1.00 (d, ^3^J_H—H_ = 6.8 Hz, 6 H, CH_3_ of ^i^Pr). ^13^C NMR: δ = 178.76, 178.22 (Ir—C), 124, 117 (N—CH—N), 91.34, 91.25 (N-CHMe_3_), 86.36, 86.28 (N—CH_3_), 52.60 (CH of COD), 38.10, 33.75, 27.99, (CH_2_ of COD), 23.5, 22.90 (CH_3_ of ^i^Pr).

### Refinement

H atoms were located from a difference Fourier map and are freely refined.

### Crystal data

**Table:**

[Rh(C_8_H_12_)(C_7_H_12_N_2_)_2_]BF_4_	*F*(000) = 1128
*M**_r_* = 546.27	*D*_x_ = 1.495 Mg m^−^^3^
Orthorhombic, *P**c**c**n*	Mo *K*α radiation, λ = 0.71073 Å
Hall symbol: -P 2ab 2ac	Cell parameters from 9624 reflections
*a* = 11.7508 (6) Å	θ = 4.2–51.7°
*b* = 11.9283 (6) Å	µ = 0.75 mm^−^^1^
*c* = 17.3129 (9) Å	*T* = 100 K
*V* = 2426.7 (2) Å^3^	Block, yellow
*Z* = 4	0.38 × 0.37 × 0.37 mm

### Data collection

**Table:**

Bruker Kappa APEXII DUO CCD diffractometer	14018 independent reflections
Radiation source: fine-focus sealed tube with Miracol optics	10241 reflections with *I* > 2σ(*I*)
graphite	*R*_int_ = 0.033
φ and ω scans	θ_max_ = 52.3°, θ_min_ = 2.9°
Absorption correction: multi-scan (*SADABS*; Sheldrick, 1996)	*h* = −26→25
*T*_min_ = 0.763, *T*_max_ = 0.771	*k* = −26→26
234794 measured reflections	*l* = −37→38

### Refinement

**Table:**

Refinement on *F*^2^	Primary atom site location: structure-invariant direct methods
Least-squares matrix: full	Secondary atom site location: difference Fourier map
*R*[*F*^2^ > 2σ(*F*^2^)] = 0.020	Hydrogen site location: difference Fourier map
*wR*(*F*^2^) = 0.059	All H-atom parameters refined
*S* = 1.13	*w* = 1/[σ^2^(*F*_o_^2^) + (0.0182*P*)^2^ + 0.6772*P*] where *P* = (*F*_o_^2^ + 2*F*_c_^2^)/3
14018 reflections	(Δ/σ)_max_ = 0.002
218 parameters	Δρ_max_ = 1.55 e Å^−^^3^
0 restraints	Δρ_min_ = −0.92 e Å^−^^3^

### Special details

**Table:**

Geometry. All e.s.d.'s (except the e.s.d. in the dihedral angle between two l.s. planes) are estimated using the full covariance matrix. The cell e.s.d.'s are taken into account individually in the estimation of e.s.d.'s in distances, angles and torsion angles; correlations between e.s.d.'s in cell parameters are only used when they are defined by crystal symmetry. An approximate (isotropic) treatment of cell e.s.d.'s is used for estimating e.s.d.'s involving l.s. planes.
Refinement. Refinement of *F*^2^ against ALL reflections. The weighted *R*-factor *wR* and goodness of fit *S* are based on *F*^2^, conventional *R*-factors *R* are based on *F*, with *F* set to zero for negative *F*^2^. The threshold expression of *F*^2^ > σ(*F*^2^) is used only for calculating *R*-factors(gt) *etc*. and is not relevant to the choice of reflections for refinement. *R*-factors based on *F*^2^ are statistically about twice as large as those based on *F*, and *R*- factors based on ALL data will be even larger.

### Fractional atomic coordinates and isotropic or equivalent isotropic displacement parameters (Å^2^)

**Table:**

	*x*	*y*	*z*	*U*_iso_*/*U*_eq_	
Rh1	0.7500	0.2500	0.507678 (3)	0.01052 (1)	
N1	0.56243 (4)	0.20105 (4)	0.38873 (3)	0.01399 (6)	
N2	0.66738 (4)	0.05648 (4)	0.40496 (3)	0.01512 (6)	
C1	0.65651 (4)	0.16534 (4)	0.42664 (3)	0.01295 (6)	
C2	0.51512 (5)	0.11598 (5)	0.34425 (4)	0.01757 (8)	
H2	0.4526 (10)	0.1269 (10)	0.3143 (7)	0.022 (3)*	
C3	0.58158 (5)	0.02502 (5)	0.35458 (4)	0.01811 (8)	
H3	0.5769 (10)	−0.0434 (10)	0.3364 (7)	0.028 (3)*	
C4	0.51719 (5)	0.31568 (5)	0.39338 (3)	0.01602 (7)	
H4	0.5633 (9)	0.3517 (9)	0.4313 (6)	0.019 (3)*	
C5	0.53212 (7)	0.37474 (6)	0.31623 (4)	0.02376 (11)	
H5A	0.4910 (11)	0.3362 (11)	0.2772 (7)	0.030 (3)*	
H5B	0.6089 (11)	0.3778 (11)	0.3028 (8)	0.031 (3)*	
H5C	0.5034 (11)	0.4517 (11)	0.3183 (8)	0.035 (3)*	
C6	0.39373 (6)	0.31435 (7)	0.41936 (5)	0.02579 (12)	
H6A	0.3478 (11)	0.2754 (11)	0.3834 (8)	0.028 (3)*	
H6B	0.3635 (11)	0.3876 (11)	0.4230 (8)	0.035 (3)*	
H6C	0.3844 (12)	0.2775 (12)	0.4678 (9)	0.033 (3)*	
C7	0.75478 (6)	−0.02030 (5)	0.43195 (4)	0.02033 (9)	
H7A	0.8005 (10)	0.0166 (10)	0.4689 (7)	0.024 (3)*	
H7B	0.7196 (11)	−0.0845 (12)	0.4540 (8)	0.031 (3)*	
H7C	0.8014 (11)	−0.0430 (11)	0.3906 (8)	0.032 (3)*	
C8	0.61291 (5)	0.22486 (5)	0.59096 (3)	0.01603 (7)	
H8	0.5433 (10)	0.2098 (10)	0.5610 (7)	0.021 (3)*	
C9	0.68830 (5)	0.13645 (5)	0.60051 (3)	0.01646 (8)	
H9	0.6691 (10)	0.0669 (10)	0.5751 (7)	0.025 (3)*	
C10	0.77717 (6)	0.12488 (6)	0.66365 (4)	0.01943 (9)	
H10A	0.7517 (9)	0.1609 (11)	0.7107 (8)	0.023 (3)*	
H10B	0.7852 (10)	0.0442 (11)	0.6775 (7)	0.026 (3)*	
C11	0.89369 (5)	0.16926 (6)	0.63829 (4)	0.01888 (9)	
H11A	0.9440 (9)	0.1813 (9)	0.6833 (7)	0.020 (2)*	
H11B	0.9335 (10)	0.1111 (10)	0.6049 (7)	0.023 (3)*	
B1	0.7500	0.7500	0.29593 (6)	0.01705 (12)	
F1	0.76439 (5)	0.84479 (5)	0.24966 (3)	0.03003 (10)	
F2	0.65419 (4)	0.76347 (4)	0.34218 (3)	0.02659 (9)	

### Atomic displacement parameters (Å^2^)

**Table:**

	*U*^11^	*U*^22^	*U*^33^	*U*^12^	*U*^13^	*U*^23^
Rh1	0.01063 (2)	0.00980 (2)	0.01114 (2)	−0.00124 (1)	0.000	0.000
N1	0.01412 (14)	0.01361 (14)	0.01424 (15)	0.00070 (11)	−0.00193 (11)	−0.00109 (12)
N2	0.01684 (16)	0.01164 (14)	0.01689 (16)	0.00010 (12)	−0.00365 (13)	−0.00144 (12)
C1	0.01345 (15)	0.01194 (15)	0.01347 (16)	−0.00035 (12)	−0.00093 (12)	−0.00044 (12)
C2	0.01794 (19)	0.01713 (19)	0.0176 (2)	−0.00031 (15)	−0.00510 (16)	−0.00262 (15)
C3	0.0211 (2)	0.01475 (18)	0.0185 (2)	−0.00131 (16)	−0.00536 (17)	−0.00293 (15)
C4	0.01615 (18)	0.01608 (18)	0.01583 (18)	0.00398 (14)	−0.00011 (14)	−0.00123 (14)
C5	0.0321 (3)	0.0195 (2)	0.0197 (2)	0.0075 (2)	0.0032 (2)	0.00306 (19)
C6	0.0185 (2)	0.0318 (3)	0.0270 (3)	0.0065 (2)	0.0043 (2)	−0.0023 (2)
C7	0.0218 (2)	0.01268 (16)	0.0265 (3)	0.00234 (17)	−0.0073 (2)	−0.00142 (16)
C8	0.01421 (17)	0.01788 (18)	0.01599 (18)	−0.00244 (14)	0.00177 (14)	−0.00026 (15)
C9	0.01790 (19)	0.01465 (17)	0.01685 (19)	−0.00334 (15)	0.00120 (15)	0.00176 (14)
C10	0.0215 (2)	0.0200 (2)	0.0168 (2)	−0.00095 (18)	−0.00002 (17)	0.00507 (17)
C11	0.0179 (2)	0.0210 (2)	0.0178 (2)	0.00064 (17)	−0.00287 (16)	0.00310 (17)
B1	0.0149 (3)	0.0145 (3)	0.0218 (3)	0.0006 (2)	0.000	0.000
F1	0.0346 (3)	0.0235 (2)	0.0320 (2)	0.00074 (18)	0.00329 (18)	0.01073 (17)
F2	0.01926 (17)	0.02301 (19)	0.0375 (3)	0.00023 (13)	0.00938 (16)	−0.00171 (16)

### Geometric parameters (Å, °)

**Table:**

Rh1—C1	2.0482 (5)	C6—H6A	0.946 (13)
Rh1—C1^i^	2.0482 (5)	C6—H6B	0.946 (14)
Rh1—C8	2.1826 (6)	C6—H6C	0.952 (15)
Rh1—C8^i^	2.1826 (6)	C7—H7A	0.944 (12)
Rh1—C9	2.2233 (6)	C7—H7B	0.950 (14)
Rh1—C9^i^	2.2233 (6)	C7—H7C	0.941 (13)
N1—C1	1.3544 (7)	C8—H8	0.985 (12)
N1—C2	1.3899 (7)	C8—C9	1.3872 (9)
N1—C4	1.4692 (7)	C8—C11^i^	1.5074 (9)
N2—C1	1.3577 (7)	C9—H9	0.965 (12)
N2—C3	1.3850 (7)	C9—C10	1.5181 (9)
N2—C7	1.4532 (8)	C10—H10A	0.968 (13)
C2—H2	0.909 (11)	C10—H10B	0.997 (13)
C2—C3	1.3487 (9)	C10—C11	1.5322 (9)
C3—H3	0.877 (12)	C11—C8^i^	1.5075 (9)
C4—H4	0.954 (11)	C11—H11A	0.989 (11)
C4—C5	1.5202 (9)	C11—H11B	1.017 (12)
C4—C6	1.5190 (9)	B1—F1	1.3960 (8)
C5—H5A	0.950 (13)	B1—F1^ii^	1.3960 (8)
C5—H5B	0.933 (13)	B1—F2	1.3908 (8)
C5—H5C	0.979 (14)	B1—F2^ii^	1.3909 (8)
			
C1—Rh1—C1^i^	93.53 (3)	C4—C6—H6A	110.8 (8)
C1—Rh1—C8	89.36 (2)	C4—C6—H6B	111.7 (8)
C1^i^—Rh1—C8^i^	89.36 (2)	C4—C6—H6C	112.0 (8)
C1—Rh1—C8^i^	156.36 (2)	H6A—C6—H6B	106.4 (11)
C1^i^—Rh1—C8	156.36 (2)	H6A—C6—H6C	106.7 (11)
C1—Rh1—C9	91.14 (2)	H6B—C6—H6C	108.9 (11)
C1^i^—Rh1—C9	166.03 (2)	N2—C7—H7A	109.0 (7)
C1^i^—Rh1—C9^i^	91.14 (2)	N2—C7—H7B	109.3 (8)
C1—Rh1—C9^i^	166.03 (2)	N2—C7—H7C	110.4 (8)
C8—Rh1—C8^i^	97.31 (3)	H7A—C7—H7B	110.6 (11)
C8—Rh1—C9	36.69 (2)	H7A—C7—H7C	108.6 (11)
C8^i^—Rh1—C9	81.19 (2)	H7B—C7—H7C	109.0 (11)
C8^i^—Rh1—C9^i^	36.69 (2)	Rh1—C8—H8	106.8 (7)
C8—Rh1—C9^i^	81.19 (2)	Rh1—C8—C9	73.25 (3)
C9—Rh1—C9^i^	87.42 (3)	Rh1—C8—C11^i^	106.38 (4)
C1—N1—C2	111.43 (5)	H8—C8—C9	117.0 (7)
C1—N1—C4	124.16 (5)	H8—C8—C11^i^	113.3 (7)
C2—N1—C4	124.41 (5)	C9—C8—C11^i^	127.23 (5)
C1—N2—C3	111.39 (5)	Rh1—C9—C8	70.06 (3)
C1—N2—C7	125.48 (5)	Rh1—C9—H9	105.7 (7)
C3—N2—C7	123.10 (5)	Rh1—C9—C10	110.59 (4)
Rh1—C1—N1	127.93 (4)	C8—C9—H9	116.7 (7)
Rh1—C1—N2	127.62 (4)	C8—C9—C10	126.46 (6)
N1—C1—N2	104.10 (4)	H9—C9—C10	114.2 (7)
N1—C2—H2	122.4 (7)	C9—C10—H10A	110.7 (7)
N1—C2—C3	106.40 (5)	C9—C10—H10B	109.0 (7)
H2—C2—C3	131.2 (7)	C9—C10—C11	112.14 (5)
N2—C3—C2	106.68 (5)	H10A—C10—H10B	104.8 (11)
N2—C3—H3	121.6 (8)	H10A—C10—C11	111.3 (7)
C2—C3—H3	131.7 (8)	H10B—C10—C11	108.6 (7)
N1—C4—H4	104.6 (7)	C8^i^—C11—C10	113.53 (5)
N1—C4—C5	109.96 (5)	C8^i^—C11—H11A	109.7 (7)
N1—C4—C6	110.61 (5)	C8^i^—C11—H11B	106.6 (7)
H4—C4—C5	109.3 (7)	C10—C11—H11A	111.0 (7)
H4—C4—C6	110.0 (7)	C10—C11—H11B	109.8 (7)
C5—C4—C6	112.04 (6)	H11A—C11—H11B	105.8 (9)
C4—C5—H5A	110.0 (8)	F1—B1—F1^ii^	109.97 (9)
C4—C5—H5B	110.4 (8)	F1—B1—F2	109.56 (3)
C4—C5—H5C	111.2 (8)	F1^ii^—B1—F2	109.02 (3)
H5A—C5—H5B	109.5 (11)	F1^ii^—B1—F2^ii^	109.56 (3)
H5A—C5—H5C	107.7 (11)	F1—B1—F2^ii^	109.02 (3)
H5B—C5—H5C	107.9 (11)	F2—B1—F2^ii^	109.70 (9)
			
C2—N1—C1—Rh1	−173.45 (4)	C2—N1—C4—C6	56.65 (8)
C2—N1—C1—N2	0.13 (6)	C1—Rh1—C8—C9	92.76 (4)
C4—N1—C1—Rh1	7.28 (8)	C1^i^—Rh1—C8—C9	−169.87 (5)
C4—N1—C1—N2	−179.14 (5)	C1^i^—Rh1—C8—C11^i^	−45.11 (7)
C3—N2—C1—Rh1	173.54 (4)	C1—Rh1—C8—C11^i^	−142.49 (4)
C3—N2—C1—N1	−0.07 (6)	C8^i^—Rh1—C8—C9	−64.50 (3)
C7—N2—C1—Rh1	−4.70 (8)	C8^i^—Rh1—C8—C11^i^	60.26 (4)
C7—N2—C1—N1	−178.31 (6)	C9^i^—Rh1—C8—C9	−97.57 (4)
C1^i^—Rh1—C1—N1	−83.65 (5)	C9—Rh1—C8—C11^i^	124.75 (6)
C1^i^—Rh1—C1—N2	104.22 (5)	C9^i^—Rh1—C8—C11^i^	27.19 (4)
C8—Rh1—C1—N1	72.88 (5)	Rh1—C8—C9—C10	101.36 (6)
C8^i^—Rh1—C1—N1	179.85 (5)	C11^i^—C8—C9—Rh1	−98.12 (6)
C8—Rh1—C1—N2	−99.26 (5)	C11^i^—C8—C9—C10	3.25 (9)
C8^i^—Rh1—C1—N2	7.72 (8)	C1—Rh1—C9—C8	−87.40 (4)
C9—Rh1—C1—N1	109.52 (5)	C1^i^—Rh1—C9—C8	163.01 (8)
C9^i^—Rh1—C1—N1	25.66 (11)	C1—Rh1—C9—C10	149.98 (4)
C9—Rh1—C1—N2	−62.61 (5)	C1^i^—Rh1—C9—C10	40.40 (11)
C9^i^—Rh1—C1—N2	−146.48 (8)	C8^i^—Rh1—C9—C8	115.05 (4)
C1—N1—C2—C3	−0.15 (7)	C8—Rh1—C9—C10	−122.61 (6)
C4—N1—C2—C3	179.12 (5)	C8^i^—Rh1—C9—C10	−7.56 (4)
N1—C2—C3—N2	0.09 (7)	C9^i^—Rh1—C9—C8	78.69 (3)
C1—N2—C3—C2	−0.01 (7)	C9^i^—Rh1—C9—C10	−43.92 (4)
C7—N2—C3—C2	178.27 (6)	Rh1—C9—C10—C11	−13.94 (7)
C1—N1—C4—C5	111.55 (6)	C8—C9—C10—C11	−93.84 (7)
C1—N1—C4—C6	−124.17 (6)	C9—C10—C11—C8^i^	39.69 (8)
C2—N1—C4—C5	−67.63 (8)		

Symmetry codes: (i) −*x*+3/2, −*y*+1/2, *z*; (ii) −*x*+3/2, −*y*+3/2, *z*.

### Hydrogen-bond geometry (Å, °)

**Table:**

*D*—H···*A*	*D*—H	H···*A*	*D*···*A*	*D*—H···*A*
C2—H2···F1^iii^	0.909 (11)	2.496 (11)	3.3975 (8)	171.4 (10)
C3—H3···F2^iv^	0.877 (12)	2.478 (12)	3.2415 (8)	145.9 (11)

Symmetry codes: (iii) *x*−1/2, −*y*+1, −*z*+1/2; (iv) *x*, *y*−1, *z*.
